# Experimental Investigation on Hover Performance of a Ducted Coaxial-Rotor UAV

**DOI:** 10.3390/s23146413

**Published:** 2023-07-14

**Authors:** Hai Li, Zaibin Chen, Hongguang Jia

**Affiliations:** 1 Changchun Institute of Optics, Fine Mechanics and Physics, Chinese Academy of Sciences, Changchun 130033, China; 13080027307@163.com (Z.C.); jiahg@ciomp.ac.cn (H.J.); 2 University of Chinese Academy of Sciences, Beijing 100049, China; 3 Chang Guang Satellite Technology Co., Ltd., Changchun 130102, China

**Keywords:** ducted coaxial-rotor system, design parameters, hover performance, aerodynamic characteristics, figure of merit

## Abstract

This paper presents experimental investigations on aerodynamic performance of a ducted coaxial-rotor system to evaluate its potential application as a small unmanned aerial vehicle (SUAV). Aimed at determining the influence of design parameters (rotor spacing, tip clearance and rotor position within the duct) on hover performance, a variety of systematic measurements for several correlative configurations (single/coaxial rotor with or without a duct) in terms of thrust and torque, as well as power, were conducted in an attempt to identify a better aerodynamic configuration. The experimental results for the coaxial-rotor system indicated that varying rotor spacing affected the thrust-sharing proportion between the two rotors, but this had no significant effect on the propulsive efficiency. The optimal H/R ratio was identified as being 0.40, due to a larger thrust and stronger stability in the case of identical rotation speeds. As for the ducted single-rotor configuration, the tip clearance played a dominant role in improving its thrust performance, especially for smaller gaps (δ≤0.015R), while the rotor position made subordinate contributions. The maximum performance was obtained with the rotor located at the P_5_ position (0.31C_d_ from the duct lip), which resulted in an enhancement of approximately 20% in power loading over the isolated single rotor. When the coaxial rotors were surrounded within the duct, the system thrust for a given power degraded with the increasing rotor spacing, which was mainly attributed to the upper rotor suffering from heavier leakage losses. And hence, the ducted coaxial-rotor system with S_1_ spacing had the best propulsion efficiency and hover performance with a figure of merit of 0.61.

## 1. Introduction

Ducted coaxial-rotor UAVs refer to a type of compact aircraft in which the counterrotating rotors are shrouded within a duct. They are commonly capable of vertical takeoff and landing (VTOL), and possess outstanding low-altitude flying and low-speed loiter performance. This is mainly attributed to their unique aerodynamic configuration that is popularly regarded as a compound integration of the coaxial rotor and the ducted rotor units. Each presents their own notable advantages over other conventional rotary crafts. Specifically, vehicles with a coaxial-rotor configuration do not require a traditional tail rotor for anti-torque that has been verified as consuming 5–20% of the total power requirement [1,2]. This is mainly due to the two contra-rotating rotors, which not only maintain the fuselage stability by offsetting the moment in flight, but also make it more compact because of smaller rotor diameter at the same disk loading. In the case of the ducted rotor configuration (involving single or coaxial rotors), the rotor unit is definitely shrouded with a duct so that it gives an enhancement of aerodynamic performance on account of the additional lift resulting from the duct. In addition to the excellent propulsion efficiency and high maneuverability, some other operational benefits can also be observed, especially in a relatively confined environment such as a high-density urban or arboreous area, i.e., structure ruggedization, anti-collision protection, noise reduction, etc. [3,4]. Compared to conventional rotorcrafts, they consequentially contribute to offering a supplementary security feature, including a lower risk of collision accidents or rotor damage for a ducted coaxial-rotor UAV when performing various missions in complicated environments [5,6].

Given the numerous potential advantages detailed above, it has become a more attractive option to design an advanced ducted coaxial-rotor UAV in the field of drones using vertical takeoff and landing at present. Not only that, the concept of ducting a coaxial-rotor system has developed rapidly; meanwhile, this has had a wide application in terms oof both military and civilian uses in recent years. They are generally designed in a range of sizes from micro to large classes, also are used to perform a variety of missions such as surveillance, reconnaissance, powerline inspection and crop protection [7]. However, it is worth noting that this novel integration configuration applied to small rotorcrafts can frequently bring about a few disadvantages such as aerodynamic issues. For example, the existence of a duct will lead to some redundant structural weight, which in turn, reduces the actual payload. The aerodynamic interference of the two rotors or between the rotor and the duct may negatively influence the propulsion efficiency of the entire system. However, the effects of tip vortices and viscosity loss at a low Reynolds number (Re < 10^5^) from the rotor blade should not be ignored either [8]. Therefore, one great challenge of developing a ducted rotor UAV is enhancing its aerodynamic performance and optimizing the aerodynamic configuration for some varying design parameters, especially at the period of preliminary design.

In recent decades, numerous scholars have made a significant effort to investigate the aerodynamic characteristics of a coaxial rotor with/without a duct via theoretical modelling, experimental investigations, numerical simulation, etc. However, there have been few consistent, universal conclusions drawn from previous articles, and those do exist are barely suited to predicting or estimating aerodynamic performance for those aircrafts designed with the typical configuration once some variations have occurred in terms of the geometry parameters. Yao Lei et al. focused on the effect of rotor spacing on the aerodynamic performance of the coaxial-rotor configuration on an MAV scale, indicated that it did result in some difference in terms of the thrust and power, by a set of experimental measurements [8]. The aerodynamic interference can be somehow beneficial to the propulsive efficiency of the coaxial system when the separation distance between the two rotors is relatively small. An experimental study was performed on a coaxial helicopter by Puneet Singh and C. Venkatesan, whose results were also validated by a theoretical model with uniform inflow [9]. They noted that the coaxial-rotor configuration consumed much more power compared with a single rotor to produce identical thrust, a reduced separation distance degraded by the interaction factor of coaxial rotors and a larger speed of rotation of the lower rotor compared with the upper rotor, contributing to the loss of thrust. Vikram Hrishikeshavan et al. conducted experimental investigations on the hover performance of a quad-shrouded-rotor MAV in terms of collective pitch, blade number and shroud diffuser length [3]. It was found that the power loading of the shrouded rotor configuration with optimal settings was obviously enhanced (approximately 40% greater) compared with the unshrouded rotor. Moreover, a larger collective pitch of the rotor within a shroud tended to be more beneficial; both the blade number and diffuser length were roughly independent of the hover performance. Xingzhu He et al. devoted time to studying the aerodynamic characteristics of a ducted fan with coaxial rotors via computational fluid dynamics (CFD). They discovered that more lift was obtained from the ducted coaxial rotors due to the duct, the lower rotor was heavily affected by the wake of the upper rotor and the effect inversely declined with the increasing rotor space [10]. Some similar conclusions were also drawn by M. Kartidjo et al. by combining experiment tests with numerical simulation in terms of a coaxial-rotor ducted-fan UAV [11], namely, that enlarging the distance of the distance between the two rotors played a dominant role in improving the thrust and reducing the yaw moment. Most of the prior studies tended to either focus on the hover performance in order to seek out an optimal configuration by a series of experimental measures (including wind tunnel tests), or adopt the numerical simulation to determine clear characteristics related to the complicated aerodynamic environments such as the tip vortex, induced inflow, mutual interference, etc. [12,13,14,15,16,17,18,19,20,21,22]. Both of the two methods above obviously require extensive effort. Though they are deemed capable of simulating a more actual situation, they do not appear to be an optimal choice in view of some constriction factors such as the time limitation of the project. Apart from in the reports of J. Gordon Leishman [23] and Puneet Singh [9], a feasible solution of theoretical calculation on the coaxial-rotor configurations has rarely been seen due to different variations in the geometry of the model, let alone a ducted coaxial-rotor system.

Aimed at a better performance or optimal configuration, more attention was paid to the effect of the geometry of rotors as well as the duct itself in the field of aerodynamics previously, but only a few finite design parameters such as the rotor spacing were involved in intensive study. However, geometric variables do not need to be taken into account at all for the specific lift components (rotor/duct) because they are already determinate after being selected initially. In this paper, a series of exhaustive experimental investigations were conducted using a customized test stand in order to determine the optimum performance of a ducted coaxial-rotor system. A wide variety of design parameters (the rotor separation distance, tip clearance, vertical position of the rotor within duct) were considered. Aside from performance measurements for the ducted coaxial system, it is worth noting that several relevant configurations including the individual single/coaxial rotor and ducted single rotor were also tested in terms of thrust, power and net torque. As is well known, the aerodynamic environment of the ducted system is more complex and disordered in comparison with the individual coaxial-rotor system. This is mainly attributed to two contributions, i.e., mutual interference between the coaxial rotors as well as some restrictions on the wake contraction in the presence of a duct; the tip vortices included. Therefore, it is fairly difficult to clearly define the aerodynamic characteristics of a ducted coaxial-rotor system. However, in the manner described above, a performance baseline could be easily obtained to draw some quantitative comparisons on the aerodynamic performance for different configurations in terms of hover capabilities, so that their aerodynamic characteristics can be further comprehended.

## 2. Aircraft Model and Some Definitions

### 2.1. Aircraft Configuration

**Figure 1:**
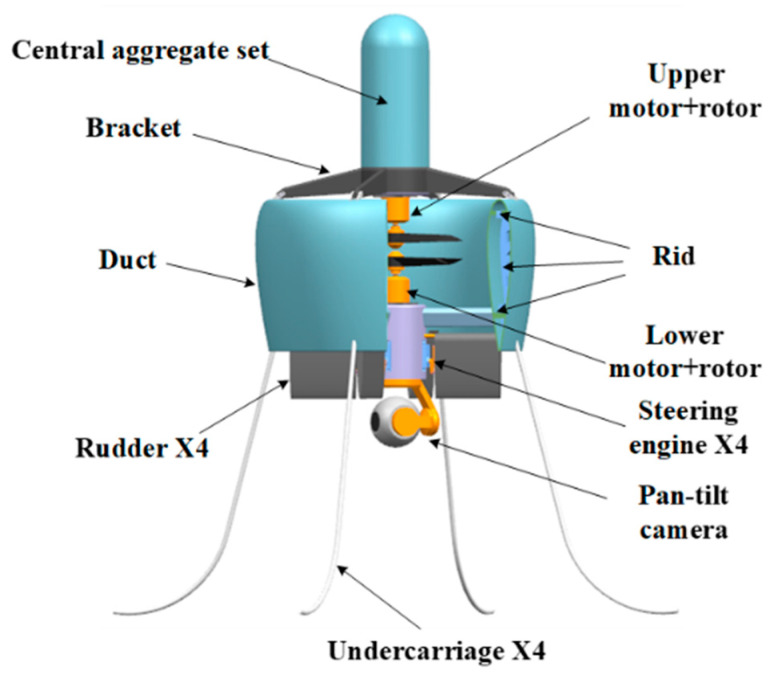
Prototype of the ducted coaxial-rotor UAV.

**Figure 2:**
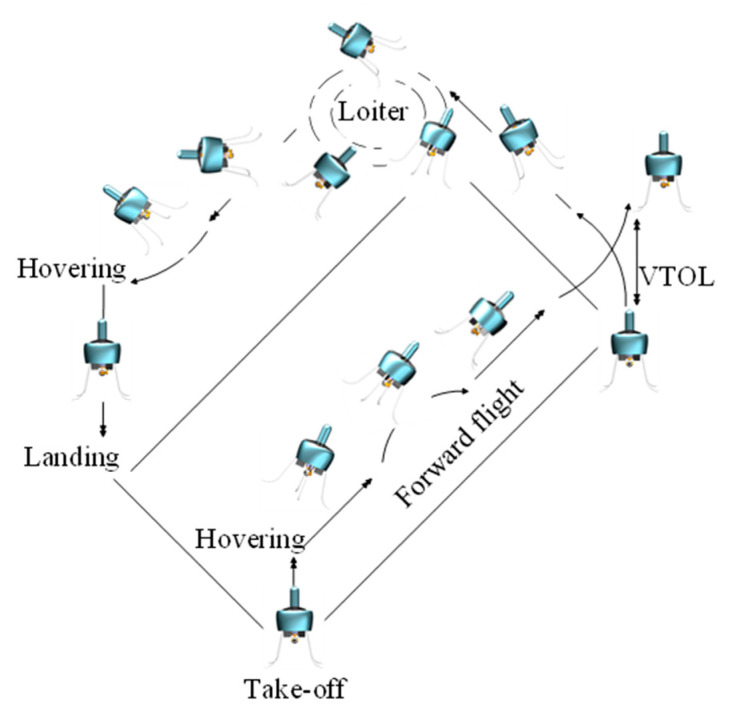
Flight profile of the designed ducted coaxial-rotor UAV.

The ducted coaxial-rotor UAV mainly consists of an annular duct and a coaxial system with two three-bladed fixed-pitch rotors (Figure 1). The geometry parameters of both the duct and rotor are shown in Table 1. The typical flight profile is displayed in Figure 2. Obviously, the aircraft not only needs to achieve vertical take-off and landing, but also needs to be capable of hover, as well as forward flight. Particularly when performing a reconnaissance mission in a complex environment, the designed ducted coaxial-rotor UAV must have an excellent hover performance, which was the focus in this paper.

### 2.2. Definitions

With regard to a ducted coaxial-rotor system, the two rotors operating vertically in the same center axis always rotate at a given rotor speed, simultaneously. In general, the rotating speed $\Omega_{u}$ and torque *Q_u_* for the upper rotor were defined to be nominally positive. On the contrary, the rotating speed $\Omega_{l}$ and torque *Q_l_* for the lower rotor were defined to be nominally negative. Given the rotor radius R, as well as the vertical rotor separation distance H, the rotor spacing can be expressed as H/R in the nondimensional form. To assess the system efficiency, it is critical to introduce a nondimensional metric, i.e., figure of merit (FOM), which indicates the ratio of effective power to input power. Normally the figure of merit η can be expressed as [24,25]:

(1) η=CT322CP=T32P2ρA

Thrust coefficient:

(2) $$C_{T}=\frac{T_{u}+T_{l}}{\rho \cdot \pi R^{2}\cdot {\left(R\Omega \cdot \frac{2\pi }{60}\right)}^{2}}=\frac{900T_{t}}{\rho \pi^{3}R^{4}\Omega^{2}}$$

Power coefficient:

(3) $$C_{P}=\frac{\Omega_{u}Q_{u}+\Omega_{l}Q_{l}}{\rho \cdot \pi R^{2}\cdot {\left(R\Omega \cdot \frac{2\pi }{60}\right)}^{3}}=\frac{27000P_{t}}{\rho \pi^{4}R^{5}\Omega^{3}}$$

In addition, the power loading is also a simple, efficient method to evaluate the propulsive efficiency in terms of the thrust generated by a certain power consumption [8]. In general, the power loading can be calculated by:

(4) $$PL=\frac{T}{P}=\frac{C_{T}}{\left(\frac{2\pi }{60}R\Omega \right)C_{P}}=\frac{30C_{T}}{\pi R\Omega C_{P}}$$

## 3. Experiment Description

### 3.1. Experimental Setup and Data Acquisition

**Figure 3:**
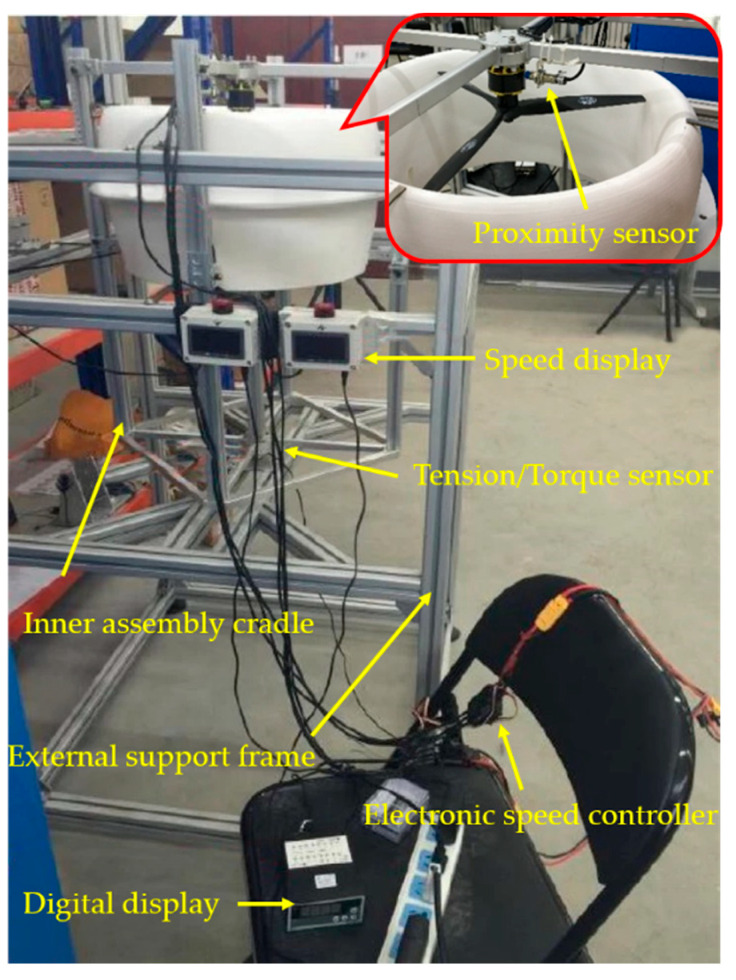
Photograph of the multifunctional test bench.

It is primarily critical for the implementation of a ducted coaxial-rotor UAV to investigate its hover efficiency affected by some dominant design parameters, i.e., the rotor spacing, rotor position and tip clearance. Therefore, a custom-designed test stand was fabricated to measure the aerodynamic performances of the full-scaled ducted coaxial rotor model, as shown in Figure 3.

**Figure 4:**
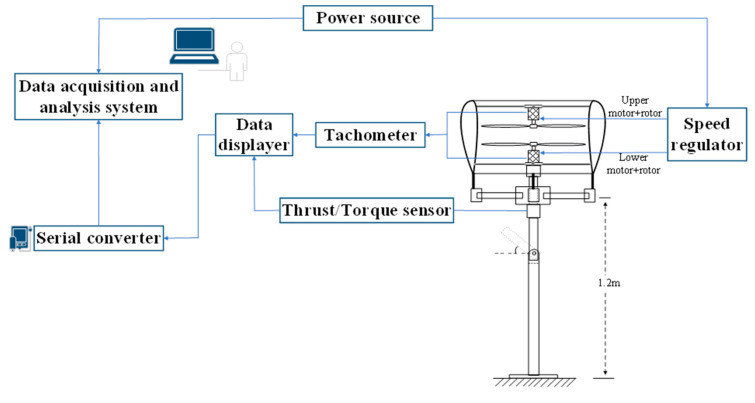
Sketch of the experimental setup.

Furthermore, the sketch of the experimental setup is displayed in Figure 4, which is conductive to understanding its basic components and the operation principle of the platform at a glance. The test stand principally consisted of two components: the inner assembly cradle as well as an external support frame. The former was mainly used to assemble the test articles involved in four relative configurations, i.e., the isolated rotor, ducted single rotor and coaxial rotor with and without a duct. Innovatively, it was convenient to achieve quick disassembly, and adjust the test portion by four sliding guides and some fixtures. For instance, the individual rotor system (including the coaxial rotor) was obtained when removing the duct from the ducted rotor configurations. As the rotor motor unit was mounted on a set of crossed support rods, measurements of the required rotor/duct position could be achieved by vertically sliding the rods up and down, to achieve the rotor spacing for variations. In addition, the test model was fixed 1.6 m above the ground to reduce any ground effect [4,8]. Specifically, the two three-bladed rotors were fixed inversely, and each of them was independently driven by one Scorpion motor (type: SII-4020-420KV) during the experiments for the coaxial-rotor system.

The rotating speed of each rotor motor unit was separately measured through an inductive proximity sensor that could sense metal objects within a range of 5 mm. A tension–compression type sensor (type: JLBM-1, accuracy: ±0.3% F.S) was mounted below the inner assembly cradle to measure the system thrust; meanwhile, its value was synchronously shown on a digital display (type: XMT808-S, accuracy: ±0.2% F.S). Similarly, the net torque derived from the two rotors with same rotational speed was measured by the torque sensor (type: JNNT-1A, accuracy: 0.1% F.S). In addition, the power consumption of each motor could be calculated by the voltage and current obtained by the measurements. During the tests, all of the quantitative values, including thrust, torque, current, voltage and rotating speed, were acquired and recorded by a data acquisition system on the computer. For the sake of observation, some measurement data (thrust, torque, rotation speed of each rotor) could even be intuitively displayed on the respective instrumentation. Furthermore, the data acquisition system simultaneously recorded and processed all of the experimental data at a rate of 300 samples per second per channel.

Notably, a reasonable method of the duct being either fixed to the inner assembly cradle or hung within the external support frame was put forward to measure the system performance separately, i.e., only the rotor loads or the entire system loads (both the rotor and duct included). In this way, the duct loads could be calculated by a simple subtraction so as to identify its influence. Obviously, it was also achievable to obtain performance measurements of the isolated single-rotor, the isolated coaxial-rotor and ducted single-rotor systems by dismantling or mounting certain relative components of the model.

### 3.2. Hover Performance Test

The chief aim of this test was to obtain the aerodynamic performances of a ducted coaxial-rotor system in hover mode in terms of thrust, torque and power consumption. Thrust was measured by the tension–compression sensor placed directly under the inner assembly cradle. As for torque, this could be measured when the thrust sensor was replaced by the torque sensor. Moreover, the power could be obtained via the current and voltage that were recorded on the data acquisition system.

It is worth noting that the torque measured in the case of the isolated/ducted coaxial-rotor configuration was the net torque of the two contra-rotating rotors. However, both of the coaxial rotors rotated at identical speeds throughout the tests. In detail, the upper rotor rotated counterclockwise, as a rule, and its torque was defined as a positive value. The opposite was true for the lower rotor, which rotated in a clockwise rotation. Due to the equal and opposite torques, a torque balance should have been achieved for the total system, in theory. However, the net torque of the coaxial system did not always remain at zero, due to the effect of aerodynamic interference, which will be verified and explained in the following discussion section of this paper.

This study was also devoted to determining the effect of aerodynamic interference on the overall performance, which universally arose from some critical parameters including the rotor spacing, rotor position within the duct, duct inlet shape, tip clearance, etc. In order to assess the influence of these parameters, a multifunctional test stand was customized and manufactured to permit some relevant variations in the tests. In summary, a series of experimental investigations on aerodynamic performance were performed using the test conditions shown in Table 2: (1) different configurations including the isolated rotor (single upper/lower rotor), coaxial rotor, ducted single rotor and ducted coaxial rotor; (2) the rotor separation distance in the case of a coaxial-rotor system (with or without a duct); and (3) tip clearance in addition to the rotor position within a duct in the longitudinal direction.

### 3.3. Test Conditions and Design Parameters

To determine the influence of these key design parameters on aerodynamic performance, a series of systematic tests for the ducted coaxial rotor and other relevant configurations of the hover mode were conducted at a laboratory (as shown in Figure 3). Primarily, the rotor speed continuously increased from 1000 to 7000 rpm at 500 rpm increments in the tests. Therefore, the Reynolds number of the blade tip correspondingly varied from 0.43 × 10^5^ to 3.01 × 10^5^, which indicated that both of the rotors were always operating at a low Reynolds number in this study.

**Figure 5:**
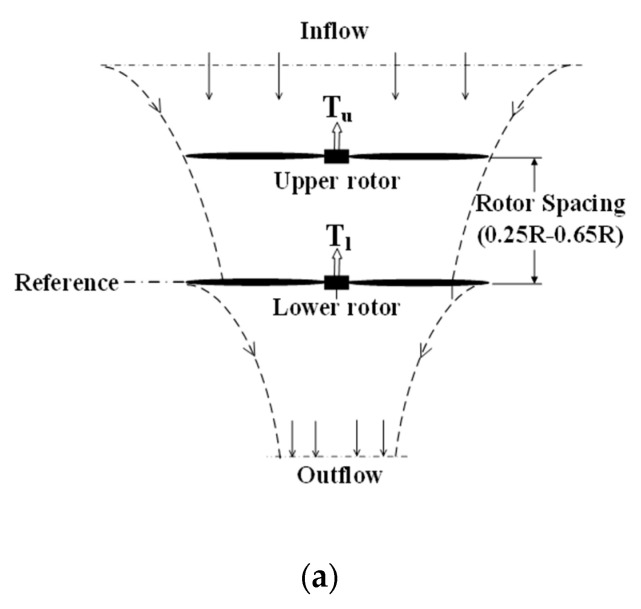

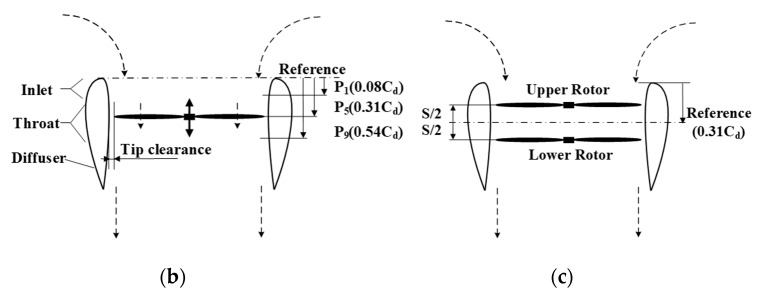
Sketches of three different aerodynamic configurations with the corresponding design parameters. (**a**) Isolated coaxial-rotor system. (**b**) Ducted a single-rotor system. (**c**) Ducted coaxial-rotor system.

In this test, the rotor spacing (S) referred to the vertical separation distance between two rotors (as shown in Figure 5a), in the case of the coaxial-rotor system. This is generally defined as a nondimensional quantity (H/R) so as to draw some relative comparisons for different configurations. As is listed in Table 2, nine different rotor spaces were specifically investigated, i.e., H/R = 0.25, 0.30, 0.35, 0.40, 0.45, 0.50, 0.55, 0.60 and 0.65.

When it comes to the unique configuration with a duct, there is no doubt that the rotor position must be one of the critical parameters. Taking the plane of the duct lip as a reference (Figure 5b), the rotor position referred to a longitudinal distance away from the reference plane when the rotor was operated within a duct. As for the ducted single-rotor system, a relatively large range varying from 0.08 C_d_ to 0.54 C_d_ was applied to study the influence of the rotor position thoroughly; herein, the Cd is the length of the duct chord. However, there were nine different sets of positions that were selected and investigated, i.e., P_1_ (0.08C_d_), P_2_ (0.13C_d_), P_3_ (0.19C_d_), P_4_ (0.25C_d_), P_5_ (0.31C_d_), P_6_ (0.37C_d_), P_7_ (0.42C_d_), P_8_ (0.48C_d_) and P_9_ (0.54C_d_). Remarkably, the tip clearance varied slightly along with the rotor position because the inner surface of the duct had a slightly small camber in this study.

With respect to the ducted coaxial-rotor system, five different rotor spacings were selected to study the effect of the duct on its aerodynamic performance by comparison with the corresponding pure coaxial-rotor system, i.e., S_1_ (0.25R), S_3_ (0.35R), S_5_ (0.45R), S_7_ (0.55R) and S_9_ (0.65R). As is shown in Figure 5c, taking the plane of P_5_ position as the reference, the upper and lower rotors were distributed symmetrically in the longitudinal direction within the duct. Lastly, it should also be noted that S and P were two shorthand notations that referred to the rotor spacing and rotor position in this paper, respectively.

## 4. Results and Discussion on Hover Performance

### 4.1. Single-Rotor Configuration

**Figure 6:**
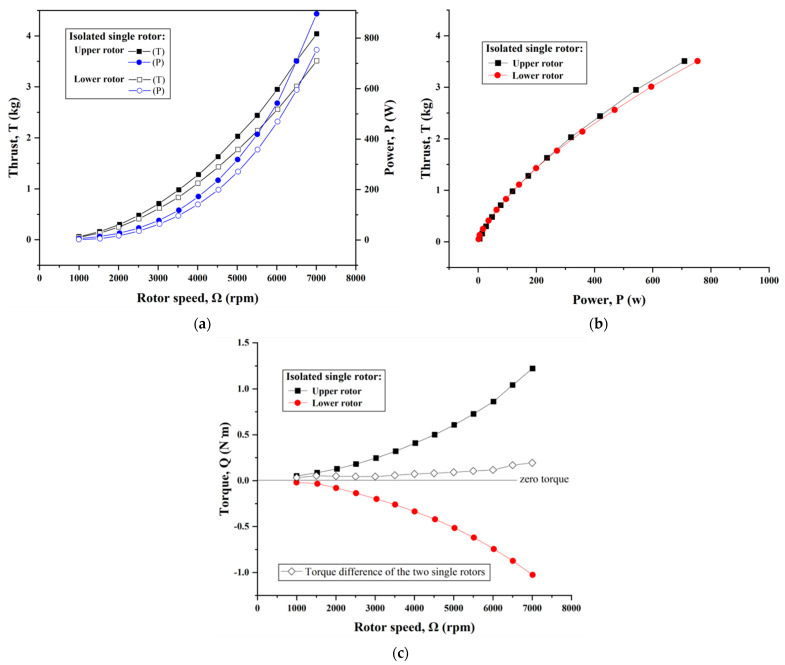
Comparison of the isolated upper and lower rotor on hover performance. (**a**) Thrust and power consumption versus rotor speed. (**b**) Thrust as functions of power. (**c**) Torque versus rotor speed.

This section presents the hover performance of the isolated single-rotor system (individual upper/lower rotor without a duct) in terms of thrust, torque and power consumption, as shown in Figure 6a–c, respectively. For the two rotors in individual operation, they always kept rotating in reverse. In addition, the rotor speed gradually increased from 1000 rpm to 7000 rpm in the tests. It is obvious that both the thrust and power rapidly increased with the rotor speed for each rotor. By fitting the experimental data, we found that both the thrust and torque produced by the upper/lower rotor proportionally varied with the square of the rotating speed; meanwhile, the power had a cube variation with the rotor speed, which was in accordance with the simple momentum theory. Notably, this outcome was obviously consistent with that in Ref. [3]. In addition, the coefficients of the fitting curve equations are listed in Table A1 in Appendix A.

Compared to the experimental results, the theoretical thrust was slightly overestimated in Figure 6a, which may be attributed to some assumptions for the aerodynamic modeling in Section 2.2. Nevertheless, this still has great significance for the predication of the rotor thrust, especially at a primary design stage, since the theoretical calculation results were approximately in line with the overall trend from the measurements. In other words, this also verified the correctness and feasibility of the theoretical model in turn because some safety allowance is fairly necessary for theoretical calculations on aerodynamic performance at the preliminary design stage for a rotary-wing UAV.

Normally, the individual upper/lower rotor with identical geometry characteristics would be considered to have the same performance, i.e., an equal thrust can be obtained by consuming the same power at a given rotor speed. However, unexpectedly, some differences in propulsion performance (thrust and power) can be seen in Figure 6a. Specifically, the upper rotor appeared to achieve a better performance than the lower, but at the expense of more power consumption. To allow for a more visual comparison, the relationships of thrust versus power consumption for the two rotors were plotted in Figure 6b. It is clear that the two single rotors possessed approximately same thrust efficiency at lower rotating speeds. However, a slight enhancement for the upper rotor in isolation occurred when the speed was above 5000 rpm. This is most likely attributed to the fact that the upper rotor that was attached downward to the support rod acted as a push unit in performance tests, but the lower rotor inversely operated as a traction unit [1]. As a result, the downwash through the lower rotor disc was slightly affected by the support arm, which lead to a slight reduction (at most 1.7%) in its power loading.

Due to counterrotating, the sign difference (positive or negative) of the measured torques can be easily observed in Figure 6c. Furthermore, the magnitude of torque produced by each rotor was nearly equal through the entire range of rotating speeds. In conclusion, the hover performance of the upper/lower rotor was fairly consistent for the single-rotor configuration. It was also verified that the experimental results were reasonably desirable in terms of their consideration of manufacture inaccuracies for the rotors, measurement errors, the influence of a low Reynolds number, etc.

### 4.2. Isolated Coaxial Rotor

**Figure 7:**
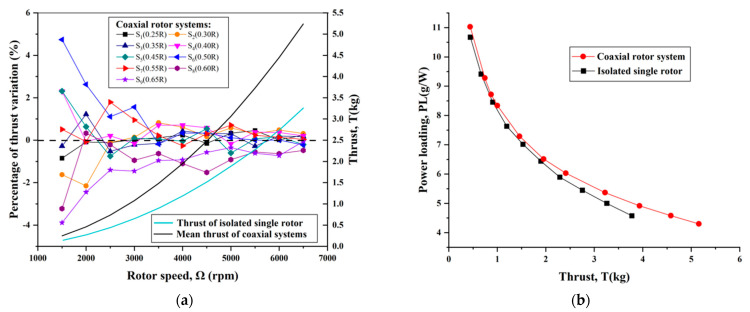
Comparison of the coaxial-rotor system and single rotor in isolation. (**a**) Thrust versus rotation speed at different spacings. (**b**) Power loading.

With regard to the performance measurements of the coaxial-rotor configuration, nine sets of nondimensional separation distances were investigated, i.e., H/R = 0.25, 0.30, 0.35, 0.40, 0.45, 0.50, 0.55, 0.60 and 0.65. Taking the average thrust performance as the datum case, the relationships of thrust variation percentage to the rotor speed at different rotor spacings are displayed in Figure 7a. This shows that the coaxial thrust significantly increased with the increasing rotor speed, and even enhanced much more rapidly in comparison with that of the individual rotor. It is also noted that the fluctuation in the thrust existed throughout the entire range of rotating speeds, though the lower RPM suffered from a more volatile phenomenon. This was mainly attributed to the viscous effect originating from the low Reynolds number [8], which was generally proportional to the rotor radium and the rotating speed. At a low RPM and induced velocity, the tip vortex stays around the rotor. When the tip vortex and vortex sheet are shed by the rotor blade, the thrust fluctuation comes into being, along with the contraction phenomenon. Therefore, it is unavoidable for a small rotorcraft to experience the problem of viscosity loss. Despite this, a solution has been put forward, i.e., surrounding the rotor with a duct, which can immensely restrain the tip vortex from developing and then decrease the thrust loss.

Apart from the above, Figure 7a also indicates that the rotor operating at S_4_, S_5_, S_6_ and S_7_ spacings achieved a better performance due to larger thrust and smaller fluctuation. This identified that the mutual interference of the two rotors seemed beneficial to the thrust performance when the rotor spacing varied between 0.40R and 0.55R, i.e., the optimum H/R ratio was within the range of 0.40–0.55 for the coaxial-rotor system. For smaller rotor spacings (H/R < 0.40), the aerodynamic interference tended to be more intense; in general, it brought about a certain induced power loss. In addition, the impingement of the wake of the upper rotor on the lower rotor became weakened with large rotor spacings (H/R > 0.55), which also had an adverse influence on the thrust efficiency.

Remarkably, the average thrust of the coaxial-rotor system was significantly inferior to the sum of the two single-rotor thrusts. As a consequence of the aerodynamic interference, the thrust loss in the coaxial-rotor system was up to approximately 14–16% in hover mode, by some calculations. Nevertheless, the coaxial system turned out to be more efficient in propulsion than the single-rotor system, as shown in Figure 7b. Specifically, the coaxial-rotor system experienced a slower decline in terms of power loading and was approximately 5.8% greater than that of the single-rotor system. This was attributed to the mutual interaction between the two rotors causing some offsets to the total power [14].

**Figure 8:**
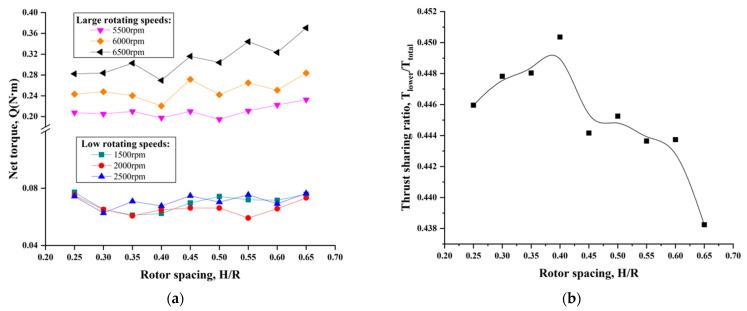
Hover performance of the coaxial-rotor system with H/R ratio. (**a**) Net torque at typical rotary speeds. (**b**) Thrust-sharing ratio for the lower rotor.

In this test, the two rotors in the coaxial-rotor system always maintained the same rotating speed, but they could not achieve a perfect torque balance. Consequently, the net torque (yaw moment) was introduced to denote the stability of the coaxial-rotor configuration in hover mode. Figure 8a showed the effect of different rotor spacings on the net torque in the coaxial-rotor system at typically low/high rotor speeds. Obviously, the net torque remained approximately constant and appeared to be independent of rotor spacing at the lower rotor speeds. However, it significantly increased at the higher rotor speeds, and a rising trend was observed with the increasing rotor spacing, especially at 6500 rpm. Moreover, it was also intriguing to observe that the smallest net torque was obtained at the rotor spacing of S_4_ (0.4R) in this test. In addition, the thrust-sharing ratio of the two rotors in the coaxial-rotor system was shown in Figure 8b. The thrust ratio of the lower rotor to the total system initially rose rapidly to max. 45.1% (at H/R = 0.40) with the increasing rotor spacing, and then slowly declined to 43.8%. This indicated the lower rotor was off-loaded; meanwhile, its thrust was far less than for the upper rotor in the coaxial-rotor configuration because the wake of the upper rotor had an enormous impingement on the lower rotor. In conclusion, this has been proven to be a simple, useful method to achieve a torque balance via the proper reduction in rotor spacing and an enhancement of the speed of the lower rotor, which can make a coaxial-rotor system more stabilized in hover mode.

**Figure 9:**
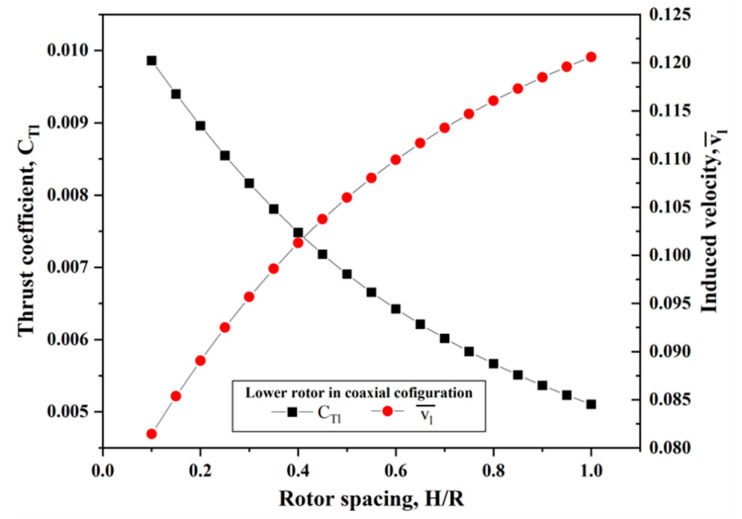
Thrust coefficient and induced velocity of the lower rotor in coaxial-rotor configuration at different spacings.

We also determined some meaningful observations in terms of the thrust coefficient and induced velocity for the lower rotor in a coaxial-rotor system by means of processing the relevant experiment data. In Figure 9, it can be observed that the induced velocity of the lower rotor improved with the increasing rotor spacing, but the thrust coefficient degraded. This can be attributed to the contracted wake of the upper rotor enlarging the inflow velocity of the lower rotor; meanwhile, its effective attack angle decreased, which further resulted in a reduction in the lift and profile drag for the lower rotor. As a consequence, both the thrust and torque produced by the lower rotor experienced a certain decrease. To sum up, the downwash from the upper rotor had a significant influence on the lower rotor, but this was not true vice versa. It is a constructive discovery that the proper enlargement of the collective pitch and the rotary speed of the lower rotor in a coaxial-rotor configuration will be beneficial to improving the thrust efficiency and reducing the yaw moment.

In conclusion, varying the rotor spacing in the coaxial-rotor system had a great impact on the thrust-sharing proportion between the two counterrotating rotors. The lower rotor generally suffered from a stronger impingement from the upper rotor wake and consequently provided a slightly smaller thrust, though the aerodynamic interference affected both rotors. Moreover, the coaxial-rotor system was more efficient in terms of propulsion due to the larger power loading as compared to the isolated single rotor, in spite of some thrust losses.

### 4.3. Ducted Single Rotor

As was mentioned previously, varying the rotor position could be achieved by sliding the support rods of a corresponding motor rotor unit to a certain location vertically within the duct for the ducted single-rotor system. Considering some structural restrictions, we adopted a resolution that the upper and lower rotor with identical geometries would be operated to approach the center gradually from either the duct inlet or trailing edge, respectively. In this way, we could afford sufficient variations on the rotor-duct position for the ducted single-rotor configuration in order to investigate the influence of shrouding a rotor with a duct. Specifically, there were five rotor locations to study the performance of the ducted upper rotor, namely P_1_, P_2_, P_3_, P_4_ and P_5_ positions, in order. Meanwhile, the P_5_, P_6_, P_7_, P_8_ and P_9_ positions corresponded to the ducted lower rotor, as shown in Table 2. Notably, both the ducted upper rotor and ducted lower rotor were measured at the P_5_ position where the rotor was 0.31C_d_ away from the duct inlet.

**Figure 10:**
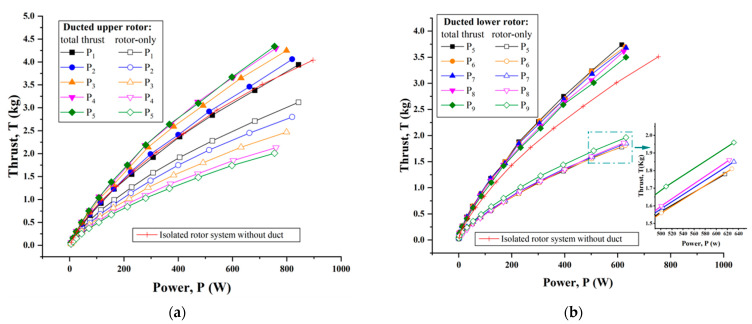
Static thrust performance of the ducted single-rotor system with variation in rotor/duct position. (**a**) Ducted upper rotor. (**b**) Ducted lower rotor.

Compared with the isolated single rotor, the ducted single-rotor system had the benefits of thrust augmentation and power decrease due to the presence of a duct. As is shown in Figure 10a for the ducted upper rotor and Figure 10b for the ducted lower rotor, two common features could be clearly observed, i.e., a greater propulsion efficiency, as well as a certain off-loading for the rotor itself. This also indicated that the duct was able to provide an additional lift and the rotor position had an effect on the system performance. When the rotor was further away from the duct lip or exit area, better thrust performance was achieved for the ducted single-rotor configuration; moreover, the optimal result occurred at the P_5_ position. This was likely attributed to the contribution of the smaller tip clearance restraining the tip vortex of the rotor and dropping the inflow loss.

**Figure 11:**
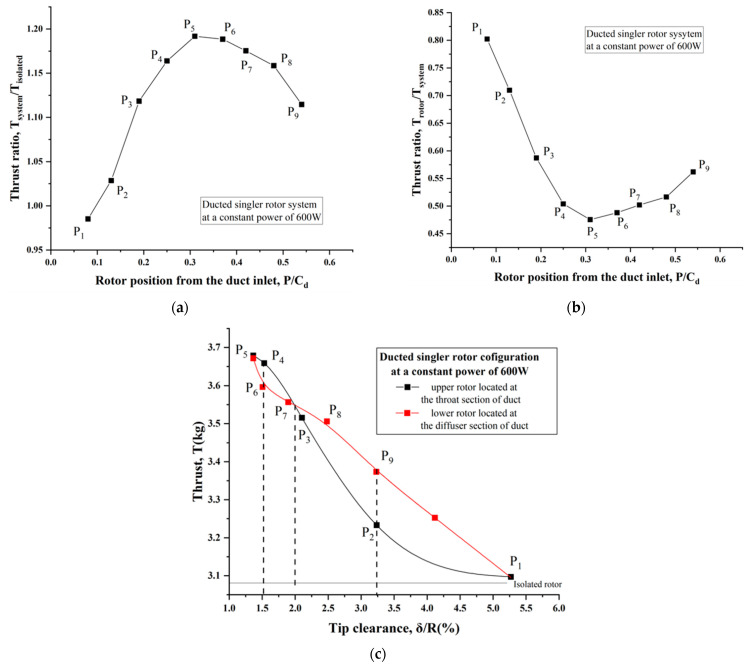
Hover performance of the ducted single rotor with variation in rotor/duct position at a constant power of 600 W. (**a**) Thrust ratio of the system to the isolated single rotor. (**b**) Thrust ratio of the rotor within duct to the system. (**c**) Influence of the rotor position and tip clearance.

To clearly illustrate the influence of the rotor duct position in detail, Figure 11a displays the thrust ratio of the overall system to the isolated single rotor at a constant power of 600 W. A higher thrust ratio indicated more gains in terms of system efficiency, namely, it would require less power to produce the same amount of thrust when the rotor was surrounded by a duct. Clearly, the best performance occurred at the P_5_ position where the thrust produced by the ducted single-rotor system was approximately 20% higher than that of the isolated single rotor. This was due to the duct playing a crucial role in terms of system performance enhancement, while the rotor position was another critical incentive. Moreover, Figure 11b also presents the thrust distribution proportion between the duct and rotor at different rotor positions. Notice that the contribution from the rotor itself to the total thrust showed a declining trend when it was further away from the duct leading or tailing edge. This was supplementary verification for the fact that the duct provided greater thrust and the rotor suffered relatively larger off-loading. Notably, the maximum proportion of the duct thrust to total thrust was up to approximately 53% when the rotor was operated at the P_5_ position (0.31C_d_).

The duct in this study was obtained by revolving the specific airfoil (NACA0018), therefore it had a slightly cambered inner surface and tapered trailing edge, with a diffuser angle of approximately 6 degrees. When varying the rotor position within the duct, it would correspondingly bring about a series of different tip clearances, though they were relatively small in comparison with the dimension of the rotor. To further study the influence of tip clearance, the measurement results at a constant power of 600 W are disclosed in Figure 11c. It is obvious that reducing the tip clearance was critical in improving the propulsive efficiency of the system, as a smaller gap tended to generate less tip losses from the leakage vortex. For this experiment, the best performance was distinctly obtained at the P_5_ location, where the minimum tip clearance (approximately 1.4%R) occurred. When the rotor was located at the throat section of the duct (for the ducted upper-rotor configuration in this test), the gains in thrust decreased more rapidly with increasing tip clearance due to the lower mass of suction flow. It was noted that the critical tip clearance was approximately 2.0% of the rotor radius. For the P_4_ and P_6_ positions with the same tip clearance, the total thrust was enhanced by approximately 16.9–18.8% compared with that of the isolated single rotor, i.e., the contribution of the rotor position variation to the performance improvement was less than 2%. For the P_2_ and P_9_ positions, there was a 4.9–9.4% increase in thrust, as the rotor was surrounded by the duct, and the corresponding increment caused by the rotor position was up to 4.5%. It was apparent that the rotor position had a negligible effect on thrust improvement when the tip clearance was below 0.02R, but this was not true for a large tip clearance (δ>0.02R). In brief, the hover performance tended to be more sensitive to the tip clearance rather than the rotor duct position.

**Figure 12:**
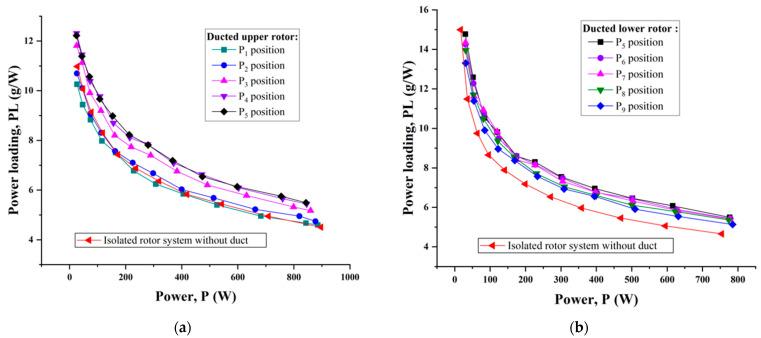
Comparison of the ducted single rotor and isolated single-rotor system on power loading at some typical rotor positions. (**a**) Ducted upper rotor. (**b**) Ducted lower rotor.

To quantify the efficiency of a ducted rotor, power loading (thrust produced per unit power) was assessed. Figure 12a,b show the power loading of the ducted upper rotor as well as the ducted lower rotor with different rotor positions, respectively. As always, the performance of the isolated rotor was selected as a baseline. Clearly, the power loading decreased somewhat more slowly when the power was more than 200 W. Compared to the respective isolated rotor, the ducted single-rotor system mostly brought about an enhancement in power loading for nearly all rotor positions. It is also worth noting that the best performance was achieved when the rotor was operated at the P4, P5 and P6 positions, where the tip clearance was within 1.5% of the rotor radius. The augmentation in thrust efficiency for a ducted single-rotor system was as much as 20% more than for the isolated rotor configuration. 

First of all, the presence of the duct augmented the inflow mass and restricted the wake contraction so that it also resulted in a certain off-loading for the rotor. However, the duct was able to offer an additional lift for the system due to the suction pressure gradient around its lip. Secondly, the tip clearance played a critical role in improving the thrust efficiency, while the rotor position had a subsidiary benefit. A smaller tip clearance (below 0.015R) tended to be more advantageous due to less leakage losses around the rotor blade tip. Lastly, the rotor operating at the P_5_ position achieved the maximum propulsion performance, i.e., the optimal location proved to be P_5_ in this test.

### 4.4. Ducted Coaxial Rotor

As was previously studied for the ducted single rotor, the P_5_ rotor position turned out to be the most beneficial, so it was reasonable to symmetrically distribute the two counterrotating rotors up and down on the basis of this position (as a midpoint) for all of the following tests involving the ducted coaxial-rotor system.

**Figure 13:**
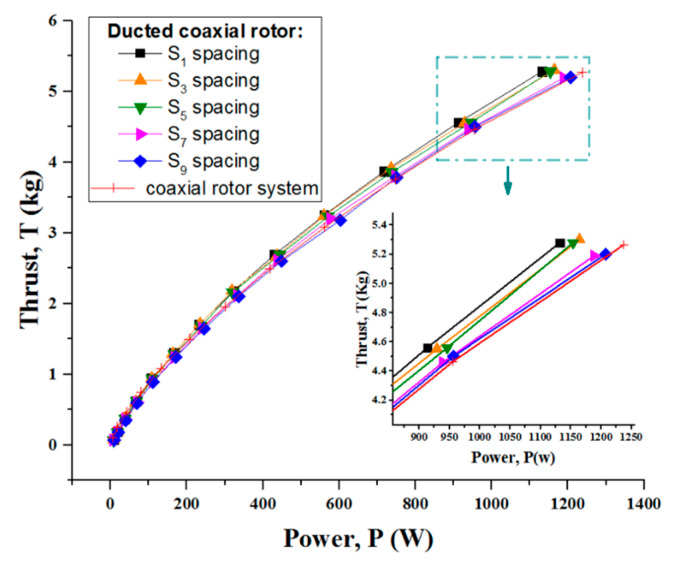
Static thrust performance of the ducted coaxial rotor at five different rotor spacings.

**Figure 14:**
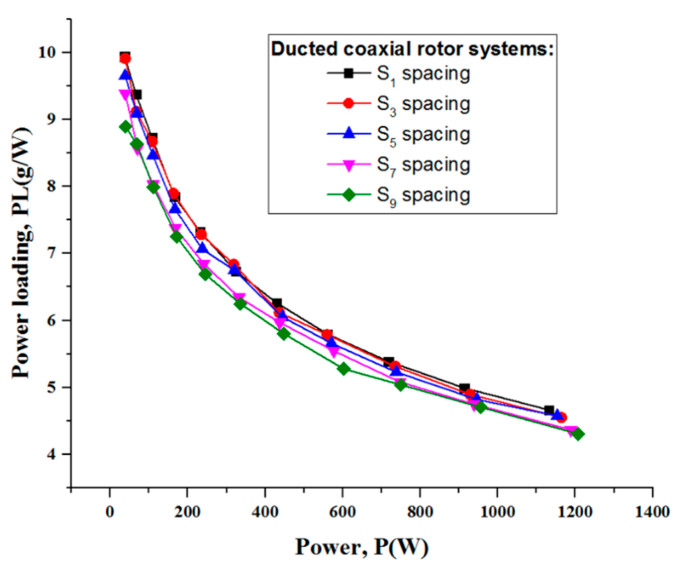
Power loading of the ducted coaxial rotor at five different rotor spacings.

Figure 13 shows the thrust variation versus power at five different rotor spacings: S_1_, S_3_, S_5_, S_7_ and S_9_. Clearly, the system thrust continued to rise with the increasing power consumption. What is more, the coaxial rotor with a duct had a greater thrust efficiency than the isolated coaxial system. It is worth noting that the rotor spacing did indeed result in some difference in the system performance. When the rotor spacing became increased, the thrust produced for a given power somewhat degraded. It is worth noting that the ducted coaxial rotor with the S_1_ rotor spacing achieved the best performance, for which power loading was almost 9% larger than for the other spacings (Figure 14).

**Figure 15:**
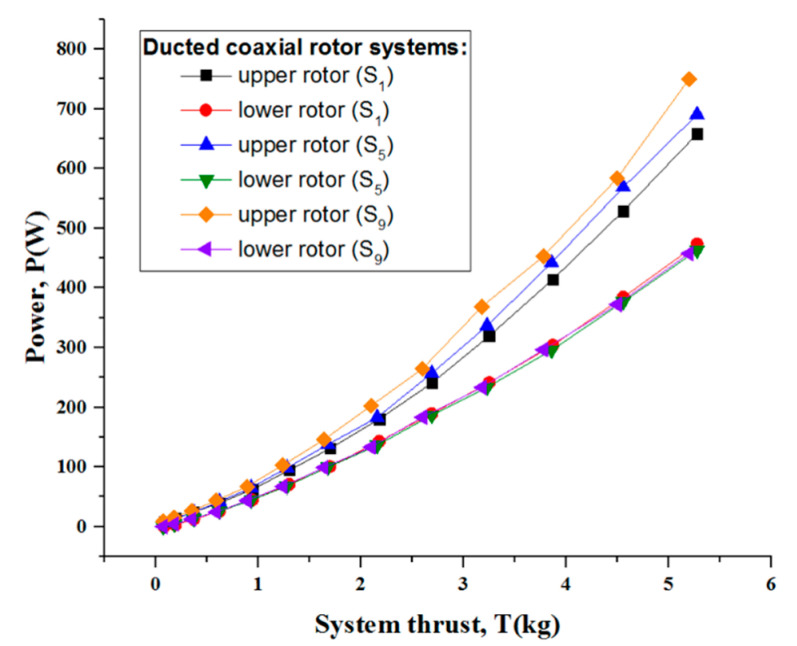
Upper and lower rotor power versus system thrust for the ducted coaxial rotor at typical rotor spacings in comparison with the respective isolated rotor.

To illustrate the impact of the duct on each of two rotors, it was necessary to determine the respective power required by the counterpart rotor at a given thrust. For simplicity, only a few representative spacings (S_1_, S_5_ and S_9_) were selected to be plotted in Figure 15 so as to make a quantitative comparison. Obviously, the power required by the lower rotor nearly remained constant for different spacings when the coaxial rotors were surrounded by the duct. We also observed that the upper rotor took up the majority of the gross power. However, the larger the rotor spacing was, the more power it consumed. This subtle difference was fairly typical and deserved intensive study. This can be put down to the existence of the duct, which restrained the upper rotor wake from contracting normally as well as stopped the lower rotor from drawing a mass of fresh air from the outer region. That is to say that almost all of the disc of the lower rotor was immersed in the slipstream of the upper rotor (the effective disc area of the lower rotor approximately identified with the inner cavity of the duct), whereas there was no sufficient mass flow through the upper rotor due to some tip vortex losses [7,11,23].

**Figure 16:**
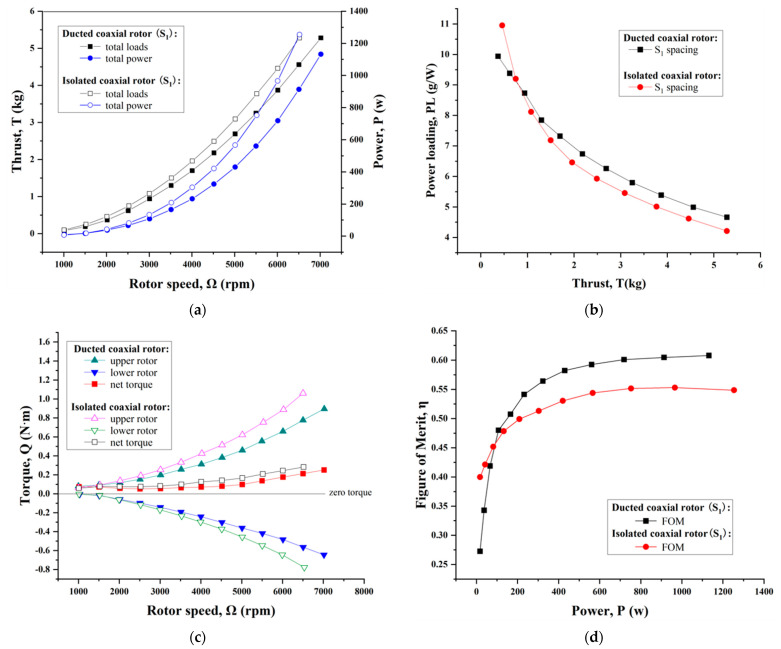
Comparison of the ducted coaxial rotor and isolated coaxial-rotor system with S_1_ spacing on the aerodynamic performance. (**a**) Thrust and power variation. (**b**) Power loading. (**c**) Torque. (**d**) Figure of merit as a function of power consumption.

Figure 16 summarizes the aerodynamic performances (including thrust, power, power loading, torque and figure of merit) for the ducted coaxial rotor with the S_1_ spacing in comparison with the isolated coaxial-rotor system. Distinctly, despite a slight reduction in thrust for the ducted coaxial-rotor system, its power consumption was immensely degraded to a much greater extent at the same rotor speed (Figure 16a). As is depicted in Figure 16b, the ducted coaxial-rotor configuration contributed to a larger power loading on the whole, i.e., it turned out to be more efficient in terms of propulsion than the coaxial-rotor system without a duct. Furthermore, each rotor achieved a smaller torque in the ducted coaxial system, in addition to the net torque (Figure 16c). This meant that large power is not necessary to counteract the yaw moment in hover flight mode. Figure 16d revealed that the ducted coaxial-rotor configuration gave a larger figure of merit (FOM), and the maximum was 0.61. It increased by approximately 12% in comparison with that of the coaxial-rotor configuration by calculation. This indicated the presence of the duct had a significant enhancement on the hover efficiency, which verified the performance benefits from another perspective.

## 5. Conclusions

A representative ducted coaxial-rotor configuration was proposed and investigated intensively by experimental investigations so as to assess its practicability for a small unmanned aerial vehicle (SUAV). As is well known, it is a challenge to draw a universal conclusion about how to achieve the optimal performance for a ducted coaxial-rotor system, in view of some complex aerodynamic issues including a mass of variations in geometry models.

The present study primarily concentrated on assessing the aerodynamic characteristics of several correlative configurations with various design parameters (the rotor spacing, rotor-duct position and tip clearance) by performance measurements. Namely, a detailed and thorough investigation on the hover performance was applied to not only the ducted coaxial-rotor configuration but also the single-rotor, coaxial-rotor and ducted single-rotor systems. This behavior was helpful in further understanding the influence of varying parameters as well as each aerodynamic component (rotor and duct) on aerodynamic performance so that the optimal configuration may be obtained.

Based on the performance measurements of the ducted coaxial rotor and three other relevant configurations, several intriguing findings and constructive conclusions were drawn as follows:

1. The thrust produced by an individual rotor acted on a quadratic function of the rotational speed; meanwhile, the power was directly proportional to the cube of it.
2. With regard to the coaxial-rotor configuration, the rotor spacing had a significant influence on the thrust-sharing proportion between two rotors rather than the thrust enhancement of the entire system. The lower rotor was heavily impinged upon by the upper rotor slipstream; in turn, it also restrained the upper rotor’s wake contraction in normal circumstances. This was due to the mutual interaction between the two rotors that offset a part of the power in some degree and contributed to a larger power loading. Notably, extending the collective pitch angle and improving the rotary speed of the lower rotor turned out to be a beneficial solution, allowing better implementation of the coaxial-rotor system.
3. For a ducted single-rotor configuration, the duct itself played a crucial role in terms of system performance enhancement, while the rotor position was another incentive. The duct not only provided a considerable additional lift but also had a great enhancement on the power loading, which was mainly attributed to the fact that the duct can effectively restrain the tip vortex from developing and then decrease the thrust loss due to the viscous effect. Furthermore, it is more beneficial to improve the thrust performance when the rotor is located away from the duct inlet and outlet. The tip clearance was more sensitive to the hover performance than the rotor duct position. A smaller tip clearance (δ≤0.015R) proved to be more beneficial for a greater thrust efficiency due to less leakage losses occurring around the rotor blade tip.
4. When the coaxial rotors were surrounded by a duct, the rotor spacing indeed made some difference to the system’s performance. Specifically, the overall propulsion efficiency somewhat degraded with a larger rotor spacing. Decreasing the rotor spacing caused the upper rotor to consume a smaller amount of power, while it barely had an influence on the lower rotor. In general, the ducted coaxial-rotor system with a smaller rotor spacing tended to achieve a better propulsion efficiency and hover performance.

Finally, individual thrust measurements of the respective rotor for the ducted/un-ducted coaxial-rotor system should be conducted in a future study so as to clearly determine the thrust contribution between two rotors. However, the method of flow visualization can also be applied in order to intuitively understand the aerodynamic characteristics including the aerodynamic interference between rotors, as well as the interaction between the rotor unit and the duct body.

## Figures and Tables

**Table 1:** Model parameters.

	Parameter	Value
Duct	Airfoil	NACA0018
	Chord	260 mm
	Lip radius	3.5 mm
	Inner diameter	386 mm
Rotor	Airfoil	NACA4415
	Diameter	380 mm
	Average chord	30 mm
	No. of blades	3
	Solidity	0.15
	Hub diameter	33 mm
	Blade angle at tip	11°
	Blade twist angle	−15°

**Table 2:** Test conditions.

Test Subject	Test Parameter	Notation and Variable
Isolated rotor	Rotor speed	1000, 1500, 2000, 2500, 3000, 3500, 4000, 4500, 5000, 5500, 6000, 6500, 7000 (rpm)
Coaxial rotors	Rotor spacing	S_1_ (0.25R), S_2_ (0.30R), S_3_ (0.35R), S_4_ (0.40R), S_5_ (0.45R), S_6_ (0.50R), S_7_ (0.55R), S_8_ (0.60R), S_9_ (0.65R)
Ducted single rotor	Rotor position Tip clearance	P_1_ (0.08C_d_), P_2_ (0.13C_d_), P_3_ (0.19C_d_), P_4_ (0.25C_d_), P_5_ (0.31C_d_), P_6_ (0.37C_d_), P_7_ (0.42C_d_), P_8_ (0.48C_d_), P_9_ (0.54C_d_)
Ducted coaxial rotor	Rotor spacing within duct	S_1_ (0.25R), S_3_ (0.35R), S_5_ (0.45R), S_7_ (0.55R), S_9_ (0.65R)

## Data Availability

Data are available from the authors upon reasonable request.

## Appendix A

For the rotors in isolation, i.e., the single upper/lower rotor, we carried out data fitting of the hover performance in terms of thrust, torque and power. The fitting curve equations for the hover performance (H_p_) are described as follows: $H_{p}=a_{0}+a_{1}\Omega +a_{2}\Omega^{2}+a_{3}\Omega^{3}$. All of the coefficients are listed in Table A1.

**Table A1:** Data fitting of the isolated single rotor.

Test Subject	H_p_	${a_{0}}_{\;}$	$a_{1}$	$a_{2}$	$a_{3}$
Upper rotor	T_u_	0.0127	−3.4912 × 10^−5^	8.7220 × 10^−8^	—
	Q_u_	0.0565	−1.8959 × 10^−5^	2.6047 × 10^−8^	—
	P_u_	−29.6813	0.0405	−1.2575 × 10^−5^	3.6524 × 10^−9^
Lower rotor	T_l_	−0.0073	−2.0441 × 10^−5^	7.4413 × 10^−8^	—
	Q_l_	−0.0062	2.5608 × 10^−6^	2.0427 × 10^−8^	—
	P_l_	−17.3511	0.0193	−5.7817 × 10^−6^	2.6638 × 10^−9^
